# Molecular Epidemiology of Influenza A/H3N2 Viruses Circulating in Mexico from 2003 to 2012

**DOI:** 10.1371/journal.pone.0102453

**Published:** 2014-07-30

**Authors:** Marina Escalera-Zamudio, Martha I. Nelson, Ana Georgina Cobián Güemes, Irma López-Martínez, Natividad Cruz-Ortiz, Miguel Iguala-Vidales, Elvia Rodríguez García, Gisela Barrera-Badillo, Jose Alberto Díaz-Quiñonez, Susana López, Carlos F. Arias, Pavel Isa, Members of Colegio de Pediatría del Estado de Veracruz

**Affiliations:** 1 Instituto de Biotecnología, Universidad Nacional Autónoma de México, Cuernavaca, Morelos, México; 2 Fogarty International Center, National Institutes of Health, Bethesda, Maryland, United States of America; 3 Instituto de Diagnóstico y Referencia Epidemiológicos, México D.F., México; Rockefeller University, United States of America

## Abstract

In this work, nineteen influenza A/H3N2 viruses isolated in Mexico between 2003 and 2012 were studied. Our findings show that different human A/H3N2 viral lineages co-circulate within a same season and can also persist locally in between different influenza seasons, increasing the chance for genetic reassortment events. A novel minor cluster was also identified, named here as Korea, that circulated worldwide during 2003. Frequently, phylogenetic characterization did not correlate with the determined antigenic identity, supporting the need for the use of molecular evolutionary tools additionally to antigenic data for the surveillance and characterization of viral diversity during each flu season. This work represents the first long-term molecular epidemiology study of influenza A/H3N2 viruses in Mexico based on the complete genomic sequences and contributes to the monitoring of evolutionary trends of A/H3N2 influenza viruses within North and Central America.

## Introduction

Influenza A virus is one of the most important pathogens of humans, responsible for 250,000 to 500,000 deaths annually and potentially millions during major pandemics [1], [2]. These single-stranded, negative-sense RNA viruses of the family *Orthomyxoviridae* cause regular seasonal epidemics and occasional global pandemics [3]. Influenza viruses are antigenically variable pathogens with a high evolutionary rate that gives them the capacity to evade the immune system and keep circulating in human populations. The two main evolutionary mechanisms that allow influenza viruses to constantly evolve and re-infect their hosts are antigenic drift and antigenic shift [3]. Antigenic drift occurs as a result of progressive accumulation of mutations that become fixed in the viral genome. Such mutations can confer minor changes in the viral proteins that may be advantageous for viral fitness, including the capacity to escape immune system recognition. During antigenic shift, an influenza A virus strain may acquire the HA segment, and possibly the NA segment as well, from an influenza virus of a different subtype, resulting in a viral strain with novel antigenic proteins [4].

During a given flu season multiple viral lineages may be introduced into a discrete population, and genetic reassortment may occur among co-circulating viruses giving place to novel viral strains with gene segments from different origins [5] [6]. Moreover, whole influenza viruses from a different animal origin (usually avian or swine) can be introduced into an immunologically naive population [3]. While it has long been known that influenza viruses circulate globally, the existence, location and determinants of a common ‘source’ population from which genetic and antigenic variants might emerge remains a topic of great debate, as does the extent of viral persistence between epidemic seasons within individual localities [7], [8], [9], [10], [11].

Phylogenetic and antigenic characterization of circulating strains has identified distinct A/H3N2 viral lineages that have circulated in humans since 1968 [12], [13]. Influenza strains for the yearly seasonal vaccine are chosen based on the antigenic characterization and prevalence studies of circulating viruses previous to the start of each influenza seasons [14]. A schematic representation of the main A/H3N2 antigenic groups circulating globally after the year 2000 is shown in Fig. S1 [15], [16], [17], [18], [19], [20], [21], [22], [23], [24], [25], [26], [27], [28].

A/H3N2 epidemiology studies have shown that the A/Panama/2007/99-like viruses circulated from 2001 until early 2003, and were then displaced by the A/Fujian/411/2002-like viruses arising in 2003 and circulating through mid-2004 [16], [17], [18]. In 2004, the A/California/07/2004-like viruses displaced the A/Fujian/411/2002-like strains and dominating until mid-2006 [18], [19], [20], [21]. Previous observations suggest that the Fujian-like viruses arose from a reassortment event between viruses closely related to the earlier A/Panama/2007/99-like strains and the later circulating A/California/07/2004 viruses [29]. In 2006, a group of viruses with an antigenically A/Wisconsin/67/2005-like HA and an M2 protein bearing the S31N mutation conferring amantadine-resistance, started circulating worldwide displacing the California-like viruses and dominated until early 2008 (2007; 2008). This group of viruses, also named the N-lineage, originated once more by a reassortment event, which most likely occurred in early 2005, generating a new lineage of adamantane resistant A/H3N2 viruses [30], [31]. The emergence of the N-lineage marked a hallmark in the rise of amantadine-resistant virus prevalence, as the global widespread and dominance of the N-lineage during 2006 season contributed to the fixation of this mutation in the subsequent circulating strains in the following years [31]. In mid-2007, the divergent A/Brisbane/10/2007-like viruses started circulating worldwide until mid-2009, followed by the A/Perth/16/2009- like viruses that dominated through early 2012 [23], [24], [25], [26], [27]. Later in 2012, the A/Victoria/361/2011-like viruses arose and circulated through 2013 [28].

In a global context, it has been proposed that A/H3N2 viruses that circulate in South-East Asia may function as a key source population for other locations, including the Americas, Europe, and Africa, where the virus typically dies out at the end of each flu season (sink – source model) [10]. However, it has been shown that viruses from outside Asia also may contribute to A/H3N2 evolution [8]. The H3N2 influenza seasonality in Mexico typically follows the northern hemisphere temperate regions winter influenza season, comprising the time period from October through the end of April, with an observable phase shift of one or two months [32]. During some seasons, active influenza transmission can be noted in tropical and subtropical areas of Mexico in mid to late July, and may continue throughout the rest of the season and overlap with the start of the winter season of the temperate areas during late November, with the season being essentially over by January [32].

Despite of its role during the H1N1 2009 influenza pandemic, there is little information on the evolution of seasonal influenza A/H3N2 viruses in Mexico. To address this issue, this work explored the molecular epidemiology of the first set of whole-genome sequences of A/H3N2 human influenza isolates that circulated in Mexico between 2003 and 2012.

## Methods

### Ethics statement and viral sample collection

The use of human clinical samples for this study was approved by the Bioethics Committee at the Instituto de Biotecnología, UNAM. Samples were collected between 2003–2009 by personnel of the Ministry of Health, under the Mexican Official Norm NOM-017-SSA2-2012 for epidemiological surveillance. Informed consent was not obtained, as the analysis of samples for pathogen is part of the mandate of clinical testing for the public health agency. The majority of samples were from Mexico City, with some being collected in other states of Mexico. The nineteen samples used in this study were chosen randomly, covering different regions and time periods (Table 1). Viruses from InDRE (Institute of Epidemiological Diagnosis and Reference of Mexico) were adapted to grow in MDCK cells, and 11 were antigenically characterized by the Centre of Disease Control (CDC, Atlanta, USA) by hemagglutination inhibition assay using the WHO influenza reagent kits. Samples were antigenically identified prior to our analysis. Four additional clinical samples from 2011 and 2012 were collected by medical doctors during private practice in Veracruz State, Mexico, after signed consent of a parent or guardian (Table 1). Viral samples from 2011-2012 were not antigenically characterized and were used directly without cell culture adaptation.

**Table 1:** Properties of A/H3N2 Mexican Isolates and reference strains used in analysis.

Isolate Name	Collection Date	Flu Season	Site	Antigenic Identity^1^	Phylogenetic Identity^2^	Accession numbers
A/Mexico/DIF2662	12/15/03	2003–2004	Mexico City	A/Korea/770/2002-like	Clade ‘B’	KJ855448-55
A/Mexico/DIF756	2/4/03	2002–2003	Mexico City	A/Korea/770/2002-like	Korea cluster	KJ855368-75
A/Mexico/DIF940	4/23/03	2002–2003	Mexico City	A/Panama/2007/99-like	Korea cluster	KJ855384-91
A/Mexico/DIF835	7/4/03	2003–2004	Mexico City	ND^3^	Korea cluster	KJ855376-83
A/Mexico/DIF2112	1/11/05	2004–2005	Mexico City	A/California/07/2004-like	N-*lineage*	KJ855400-7
A/Mexico/DIF2160	1/11/05	2004–2005	Mexico City	A/California/07/2004-like	N-*lineage*	KJ855408-15
A/Mexico/TLA2227	9/11/05	2005–2006	Tlaxcala	A/Wisconsin/67/2005-like	N-*lineage*	KJ855472-79
A/Mexico/DIF2246	9/11/05	2005–2006	Mexico City	A/Wisconsin/67/2005-like	N-*lineage*	KJ855416-23
A/Mexico/QUE2270	8/11/05	2005–2006	Queretaro	ND	N-*lineage*	KJ855424-31
A/Mexico/DIF2601	11/18/05	2005–2006	Mexico City	ND	N-*lineage*	KJ855432-39
A/Mexico/DIF29	1/16/06	2005–2006	Mexico City	A/Wisconsin/67/2005-like	N-*lineage*	KJ855360-7
A/Mexico/MEX2640	11/22/05	2005–2006	Edomex	ND	Brisbane cluster	KJ855440-7
A/Mexico/NAY2090	4/14/07	2006–2007	Nayarit	A/Brisbane/10/2007-like	Brisbane cluster	KJ855392-9
A/Mexico/JAL25206	5/22/09	2008–2009	Jalisco	A/Perth/16/2009-like	Brisbane cluster	KJ855456-463
A/Mexico/JAL25216	5/22/09	2008–2009	Jalisco	A/Perth/16/2009-like	Brisbane cluster	KJ855464-71
A/Mexico/VER40	12/1/11	2011–2012	Veracruz	ND	Victoria cluster	KJ855328-35
A/Mexico/VER60	12/24/11	2011–2012	Veracruz	ND	Victoria cluster	KJ855336-43
A/Mexico/VER59	9/12/11	2011–2012	Veracruz	ND	Victoria cluster	KJ855344-51
A/Mexico/VER58	1/17/12	2011–2012	Veracruz	ND	Victoria cluster	KJ85552-59
**Reference strains**						
A/Panama/2007/1999	1999		Panama	A/Panama/2007/99-like	Panama cluster	DQ508862-9^5^
A/Fujian/411/2002^4^	8/11/02	2002–2003	China	A/Korea/770/2002-like	Fujian cluster	EF541397, CY112935
A/Korea/770/2002^4^	12/2/02	2001–2002	South Korea	A/Korea/770/2002-like	Korea cluster	EF473481
A/California/7/2004	9/16/04	2004–2005	USA	A/California/07/2004-like	California cluster	CY114379, CY114380, CY047444,
						CY031795, CY047417, EU146841,
						CY031796
A/Wisconsin/67/2005	8/31/08	2008–2009	USA	A/Wisconsin/67/2005-like	N-lineage	CY034116-23
A/Brisbane/10/2007	2/6/07	2006–2007	Australia	A/Brisbane/10/2007-like	Brisbane cluster	CY035022-9
A/Perth/16/2009	4/7/09	2008–2009	Australia	A/Perth/16/2009-like	Perth cluster	GQ293081, GQ293082
A/Victoria/361/2011	2011		Australia	A/Victoria/361/2011-like	Victoria cluster	KC306165

1 As determined by standard haemagglutination inhibition assay.

2 As determined in this work.

3 Not determined.

4 A/Korea/770/2002 was determined as antigenically equivalent to the A/Fujian/411/02 vaccine strain [48], [49].

5 Accession numbers of reference strain segments used in analysis.

### Viral RNA extraction and whole genome amplification

Before extracting viral nucleic acids, external or host DNA was removed by treatment with Turbo DNAse (Ambion). Total sample RNA was extracted with the PureLink Viral DNA/RNA kit (Invitrogen), using linear acrylamide as a carrier. Whole influenza genome amplification was done by multi-segment RT-PCR using a set of universal primers, MBTuni-12 (5′ ACGCGTGATCAGCAAAAGCAGG 3′) and MBTuni-13 (5′ ACGCGTGATCAGTAGAAACAAGG 3′) with the SuperScript III one-step RT-PCR platinum Taq HiFi kit (Invitrogen), as described previously [33]. All RT-PCR products were purified and concentrated by DNA clean & concentrator kit (ZYMO) and visualized in 1.5% agarose gels.

### Library preparation and sequencing

PCR products from whole genome RT-PCR were used as input material for preparation of sequencing libraries. DNA was sheared to approximately 300 base pairs by treatment with NEBNext Fragmentase (New England Biolabs) for 15 min at 37°C. Sheared DNA was purified and concentrated by DNA clean & concentrator kit (ZYMO) and used for preparation of the libraries using Illumina's Genomic DNA Sample Preparation kit with Multiplex Sample Preparation Oligo kit, as described by the manufacturer.

High throughput sequencing was performed at the Next-generation sequencing core facility located at the Instituto de Biotecnología, UNAM in Cuernavaca, Morelos. Libraries of approximately 350 nucleotides were used to generate sequencing clusters, followed by 45 or 66 cycles of single base pair extensions in the Genome Analyzer IIx sequencer (Illumina, San Diego, CA), which was followed by multiplex barcode acquisition. Computational analysis was performed using the computational cluster at the Instituto de Biotecnología, UNAM. Image analysis was carried out using Genome Analyzer Pipeline Version 1.4. All reads were quality filtered, and aligned to the reference genome A/H3N2/New York/392/2004 (GenBank accession numbers: CY002071, CY002070, CY002069, CY002064, CY002067, CY002066, CY002065, CY002068) by SMALT version 0.7.0.1 [34] and a consensus sequence was called by SAMtools version 0.1.18 [35]. To allow sequence variability, two separate alignment rounds were done. In the first round, 15 and 20 mismatches were permitted within the 46 or 65 nucleotide long reads respectively, while during the second alignment round only 5 mismatches were allowed using as a reference the consensus sequence previously generated during the first mapping round. Any reads that did not match the reference genome were not considered for further analysis.

### Phylogenetic analysis

Complete influenza A/H3N2 viral genomes from North America collected from 1998 to 2013 available on the Influenza Virus Resource-NCBI Database were downloaded from the Influenza Virus Resource as of March 2013. Dataset was limited to north hemisphere viruses following the sink source model, in which hypothetically, viruses circulating in Mexico are seeded from the northern hemisphere at the beginning of the season. Viruses from the database were chosen according to their year of collection (from 1999 to 2013) and whole genome availability. Data sets for each viral gene segment were then obtained and curated by removing redundant sequences. When available, sequences for the antigenic defining reference strains selected for vaccination for the flu seasons from 1999-2013 described in literature and by the CDC information sheets were added to each dataset (Fig. S1 and Table 1) [15], [16], [17], [18], [19], [20], [21], [22], [23], [24], [25], [26], [27], [28]. Congruent to the information available on influenza seasonality in Mexico, the following criteria were chosen for assigning seasonality of our isolates: viruses collected from January to June correspond to the previous season from year of collection (e.g. ‘A/Mexico/1/6/99’ would belong to the 1998–1999 season), while viruses from July to December correspond to the following season (e.g. ‘A/Mexico/1/7/99’ would belong to the 1999–2000 season).

The phylogenetic clusters found in this work were labeled according to the presence of antigenic reference strains (Table 1 and Fig. S1), when available. Given this, the Panama cluster corresponds to the Panama-clustering viruses, the Fujian cluster to the Fujian-clustering viruses, the Korea cluster to the Korea-clustering viruses, the California cluster to the California-clustering viruses, the N-lineage to the Wisconsin-clustering viruses, the Brisbane cluster to the Brisbane-clustering viruses, the Perth cluster to the Perth-clustering viruses, and finally the Victoria cluster to the Victoria-clustering viruses.

To define the viral clusters, all eight individual gene trees were built. Data sets were individually aligned using MUSCLE [36] implemented in SeaView [37]. For the phylogenetic analysis, a total of 756 sequences were used for PB2, 759 for PB1, 752 for PA, 758 for HA, 627 for NP, 669 for NA, 461 for the M, and 414 for NS gene. Phylogenetic analysis was performed under the maximum likelihood criteria with PhyML 3.0 [38], using GTR+G model with aLRT (SH-like) for branch support. Clusters were defined by high branch support values (only values ≥0.7 were considered), by long branches separating each cluster, and when available, by the presence of reference strains. To avoid underestimating the role of genetic reassortment events, midpoint method was chosen for rooting all trees, as it still allows making inferences about the relatedness of clusters. However, if the evolutionary rates are constant (as expected) the root should also represent the ancestral point of the tree. For HA and NA trees, branches were annotated and shaded according to influenza season, as determined by virus collection dates (considering a complete season from July to June of the following year). For the rest of viral gene trees (PB2, PB1, PA, NP, M and NS), branches were annotated and colored according strain origin (blue for Mexican isolates, red for reference strains and black for the rest of the viruses).

Finally, for the supporting phylogenetic analysis of A/H3N2 viruses circulating worldwide from 2002 to 2004, all sequences for the HA and NA segments available in Influenza Virus Resource-NCBI Database as of December 2013 were downloaded. HA and NA datasets were curated by removing redundant sequences and further used for phylogenetic analysis, as described previously. A total of 804 sequences for HA and 660 for NA were used for the phylogenetic analysis.

To further determine mutations in the previously described receptor binding and antigenic sites of the HA gene and in the amantadine resistance sites of the M gene [13], [30], [39], [40], we manually searched for specific mutations on the sequence alignments. For the HA protein, HA1 numbering was used to detect the receptor binding sites, while HA2 numbering sites (+16 from amino acid one in coding sequence) was used to assign antigenic sites [39], [40]. The HA1 and HA2 numbering refers to the two polypeptides (HA1 and HA2) products resulting of the proteolytic cleavage of the single polypeptide precursor (HA0). The five major antigenic sites in the HA protein have been mapped on the HA1 peptide [40], [41]. For clarity purposes, all amino acid positions in the text are given in HA1 numbering, which correspond to the ORF of HA gene.

## Results

To study the evolution of A/H3N2 influenza viruses circulating in Mexico, the complete genomic sequences of 19 isolates collected between 2003 and 2012 were used for phylogenetic analysis (Table 1). All sequences were deposited into GenBank, accession numbers KJ855328-KJ855479. Unfortunately, the number of samples obtained for this work was limited. Due to the small sample size used, it is unlikely that the complete H3N2 viral diversity in Mexico from 2000–2013 is represented. However, the isolates used represent random samples taken in places were influenza outbreaks were detected during the peaks of influenza seasons from 2000–2013. Therefore, the low number of viral isolates in this work could still represent the distribution and incidence of the major viral groups circulating in the collection areas at the time of the sampling.

**Figure 1:**
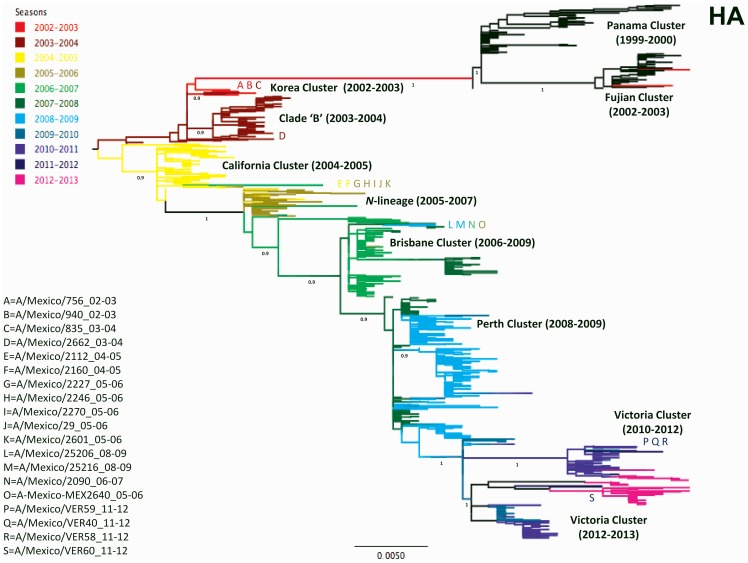
Phylogenetic analysis based on the coding sequence of the HA gene of 19 samples isolated in Mexico from 2003 to 2012. The tree was built using the ML criteria with a background of 758 selected human A/H3N2 influenza viruses from North America. Support values were determined by aLRT and only values ≥0.70 are shown for significant nodes. The tree is mid-point rooted for purposes of clarity, and all horizontal branches are drawn to scale. Different coloring in branches indicate seasonality, as established by virus collection dates (from January to June, and from July to December, respectively). Viruses from before the 2002–2003 season are shown black. The position of the Mexican isolates in the tree is indicated by coding letters, as depicted in the left portion of figure.

**Figure 2:**
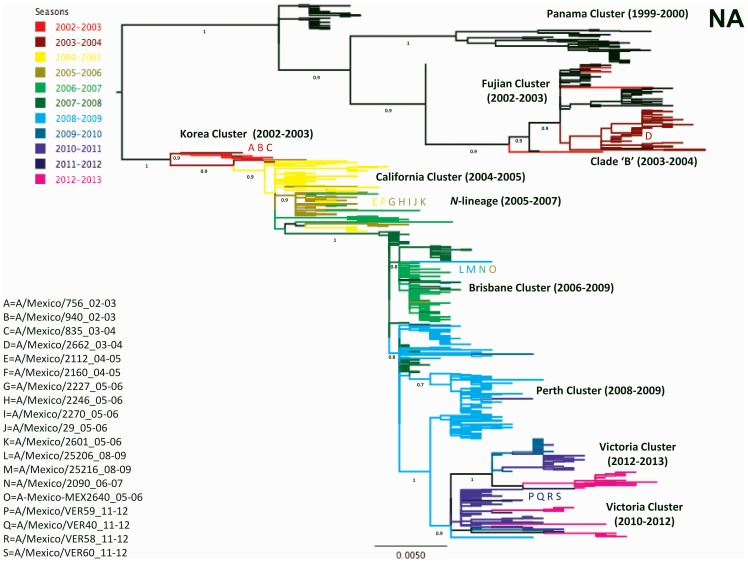
Phylogenetic analysis based on the coding sequence of the NA gene of 19 samples isolated in Mexico from 2003 to 2012. The tree was built using the ML criteria with a background of 669 selected human A/H3N2 influenza viruses from North America. Support values were determined by aLRT and only values ≥0.70 are shown for significant nodes. The tree is mid-point rooted for purposes of clarity, and all horizontal branches are drawn to scale. Different coloring in branches indicate seasonality, as established by virus collection dates (from January to June, and from July to December, respectively). Viruses from before the 2002–2003 season are shown black. The position of the Mexican isolates in the tree is indicated by coding letters, as depicted in the left portion of figure.

The HA and NA trees obtained showed seven phylogenetic clusters matching previously defined antigenic groups (Panama, Fujian, California, N-lineage, Brisbane, Perth and Victoria) (Figs. 1 and 2). The clade ‘B’ phylogenetic cluster with no matching antigenic strain was also detected. The clade ‘B’ has been characterized as a minor ancestral cluster formed by viruses from 2003–2004 closely related to the A/Panama/2007/99-like strains [29]. Further, a novel cluster, termed here Korea, was identified (Figs. 1 and 2).

The majority of the observed clusters were also evident in the PB2, PB1, PA, and NP gene trees (Figs. S2, S3, S4, S5), and to a lesser extent in the M and NS gene trees, which are not fully resolved due to the lower phylogenetic signal associated with the reduced variability in these gene segments (Figs. S6 and S7). The Mexican isolates used in this study grouped within five of the observed phylogenetic clusters: the new Korea cluster, the clade ‘B’, the N-lineage, Brisbane, and Victoria (Figs. 1 and 2, Table 1).

### The Korea cluster

The newly observed cluster was provisionally named in this study Korea (as the antigenic strain A/Korea/770/2002 groups within this cluster in the HA gene tree), and is also observed in other gene trees (PB2, PB1, PA and NP) (Figs. S2, S3, S4, S5). Our phylogenetic analysis shows that the Korea viruses have a HA and PB1 closely related to ‘old’ viruses belonging to the Panama and Fujian clusters and to the Clade ‘B’ (Fig. 1 and Fig. S3); in an HA tree, the Korea-cluster is positioned close to the clade ‘B’, while for the PB1 tree it is positioned outside the Clade ‘B’ and the Panama and Fujian clusters. On the other hand, for the NA, PB2, PA and NP gene trees, the Korea cluster is basal to the main tree trunk from which strains collected after 2004 diverge (Fig. 2, and Figs. S2, S4 and S5); in the NA and PB2 trees, the Korea cluster is closely related to the California viruses, while in the PA and NP trees it is close to the N-lineage. In the M tree, the Korea cluster is located close to the Brisbane cluster (Fig. S6), while in the NS tree the clusters cannot be defined (Fig. S7). These findings suggest a complex reassortment that gave origin to the Korea cluster viruses. Because the M gene is highly conserved, a discrete distribution of viruses according to their date of collection is not clearly visible on the M tree. Therefore the position of the Korea cluster close could be biased by the sequence similarity and does not necessarily reflect a close phylogenetic relationship between the Brisbane and Korea clusters.

Given the co-circulation of the Fujian, Korea and California clusters from 2002 to 2004, their phylogenetic differences and geographical distribution were analyzed in more detail. For this purpose, the trees were constructed for the HA and NA segments from A/H3N2 human viruses circulating worldwide from 2002 to 2004. It was observed that the Korea, Fujian, California and clade ‘B’ form defined clusters sustained by high support values both in the HA and NA trees (Figs. S8 and S9).

The Korea cluster is formed by viruses from the 2002–2003 season, including three Mexican isolates sequenced in this study (A/Mexico/DIF756/2003, A/Mexico/DIF835/2003 and A/Mexico/DIF940/2003), three other Mexican viruses characterized previously (A/Yucatan/ME6057/2003, A/Mexico/102/2003, A/Mexico/736/2003), and other viruses coming mostly from North Hemisphere indicating a global circulation of this group (Figs. S8 and S9).

### Persistence, co-circulation and temporal variations in seasonality among the Mexican A/H3N2 viruses

For the viruses collected after 2003, it was observed that seven Mexican isolates from 2005 and 2006 (A/Mexico/DIF2112/2005, A/Mexico/DIF2160/2005, A/Mexico/TLA2227/2005, A/Mexico/DIF2246/2005, A/Mexico/QUE2270/2005, A/Mexico/DIF2601/2005, A/Mexico/DIF29/2006) grouped consistently within the N-lineage (Figs. 1 and 2; Figs. S2, S3, S4, S5, S6, S7), suggesting possible local persistence of strains. However, given the low number of viruses used in this study and because the Mexican viruses observed within this lineage belong to two consecutive seasons (2004–2005 and 2005–2006), this observation cannot be fully supported.

Possible local persistence was also observed in the Brisbane cluster, containing isolates with collection dates from 2005 (A/Mexico/MEX2640/2005), 2007 (isolate A/Mexico/NAY2090/2007) and 2009 (isolates A/Mexico/JAL25206/2009 and A/Mexico/JAL25216/2009). In this case the isolates within the Brisbane cluster were collected within a span of years, moreover, there are two different isolates within the clade for the year 2009 (A/Mexico/JAL25206/2009 and A/Mexico/JAL25216/2009). These results also point out to the circulation of two distinct viral lineages in Mexico during the 2005–2006 season: the N-lineage and Brisbane cluster (Table 1).

Regarding the seasonality pattern observed in Mexico, the dates of collections of the viruses used in this work span between September and May (with exception of only two viruses, A/Mexico/DIF835/2003 and A/Mexico/QUE2270/2005 collected in July and August, respectively) (Table 1). Overall, the pattern observed is the expected due to the geographical position of Mexico, with a delay of two months in the duration of the season. It is of interest that some Mexican isolates seemed to be ahead or behind the rest of the North American viral diversity. In this sense, isolate A/Mexico/DIF835//2003 collected during the 2003–2004 influenza season grouped with viruses that circulated during 2002–2003 (Figs. 1 and 2). Similarly, samples A/Mexico/JAL25206/2009 and A/Mexico/JAL25216/2009 from the 2008–2009 season clustered with isolates from 2006–2007 and 2007–2008, once again indicating the persistence of this lineage during two subsequent seasons in Mexico (Figs. 1 and 2). On the other hand, isolates A/Mexico/DIF2112/2005 and A/Mexico/DIF2160/2005 from the 2004–2005 season grouped within the N-lineage together with samples from 2005–2006, while isolate A/Mexico/DIF2640/2006 from the 2005–2006 season group with viruses from 2006–2007 and 2008–2009, suggesting again an early circulation (Figs. 1 and 2). It was further observed that isolates A/Mexico/VER59/2011, A/Mexico/VER40/2011 and A/Mexico/VER58/2012 grouped in most of the gene trees with samples from the 2010–2011 season (Fig. 1, Figs. S2, S3, S6, and S7), while the sample A/Mexico/VER60/2011 localized close to viruses from 2012–2013, seeming to go ahead than the rest of the Mexican isolates from the same year. However, this pattern was not observed for the NA, PA and NP gene trees, as there is no clear segregation according to seasonality for the Victoria cluster in these gene trees (Fig. 2, Figs. S4 and S5).

### Phylogenetic/Antigenic Characterization and Variations in the HA gene

For several of the Mexican isolates used in this study, the phylogenetic characterization did not correlate with their antigenic identity. For the viruses belonging to the Korea cluster, one Mexican isolate (A/Mexico/DIF756/2003) was determined as A/Korea/770/2002-like, while Isolate A/Mexico/DIF940/2003 was found to belong to the A/Panama/2007/99-like antigenic group (Table 1). Interestingly, isolate A/Mexico/DIF2662/2003 that grouped within the clade ‘B’ in the phylogenetic analysis was characterized antigenically as being A/Korea/770/2002-like (Table 1, Figs. 1 and 2; Figs. S2, S3, S4, S5, S6, S7). This observation provides additional evidence for the close similarities in the HA protein of the clade ‘B’ and the Korea cluster (Tables 1, Fig. 1).

For the isolates belonging to the N-lineage, only three out of seven viruses were antigenically determined as A/Wisconsin/67/2005-like (A/Mexico/TLA2227/2005, A/Mexico/DIF2246/2005, A/Mexico/DIF29/2006), while two other were characterized as A/California/07/2004-like viruses (A/Mexico/DIF2112/2005 and A/Mexico/DIF2160/2005) (Table 1). For the Brisbane cluster, only one out of four viruses (A/Mexico/NAY2090/2007) was antigenically determined as Brisbane-like, while other two were found to be A/Perth/16/2009-like (A/Mexico/JAL25206/2009, A/Mexico/JAL25216/2009) (Table 1).

We further analyzed specific mutations in the receptor binding and antigenic sites of the HA protein sequences of our isolates and reference strains. We found that there is a clear distinction in the receptor binding sites of the Panama and Korea clusters (circulating from 2003 and before) compared to viruses circulating after the 2004–2005 season (California, Wisconsin, Brisbane, Perth, and Victoria clusters). It is of interest that the A/Fujian/411/2002 strain shares receptor-binding amino acids with A/California/7/2004 reference strain and not with the A/Korea/770/2002 strain, (Table 2) although these two viruses had been characterized as antigenically equivalent [40], [41].

**Table 2:** Mutations in the HA and M2 proteins.

	Receptor Binding Sites (HA)^1^							Antigenic Sites (HA)^1^						M2^2^
Strain Name	190	193	222	225	226	227	A (156–162)	B (171–176)^3^	B (204–214)^3^	C (292–297)	D (220–236)	E (186–190)^3^	E (275–281)^3^	31 (S/N)
A/Panama/2007/1999	D	***S*** 4	***W***	***G***	***V***	***S***	KRRSN***K***S	***HQ***LK***Y***K	D***S***DQI***SI***YAQ***S***	KCNSEC	VSTKRSQQTVIPNIGS***S***	NNEKF	KIRSGKS	***S***
A/Korea/770/2002	D	***S***	R	***D***	***V***	***S***	KRRSN***K***S	THLK***Y***K	D***S***DQI***S***LYAQA	KCNSEC	VSTKRSQQTVIPNIGSR	NNEKF	KIRSGKS	***S***
A/Fujian/411/2002	D	***S***	R	***D***	I	P	KRRSNNS	THLK***Y***K	***N***NDQI***S***LY***T***QA	KCNSEC	VSTKRSQQTVIPNIGSR	NNEKF	KIRSGKS	***S***
A/Mexico/DIF2662/2003	D	***S***	R	***D***	***V***	***S***	KRRSN***K***S	THLK***Y***K	D***S***DQI***S***LYAQA	KCNSEC	VSTKRSQQTVIPNIGSR	NNEKF	KIRSGKS	***S***
A/Mexico/DIF756/2003	D	***S***	R	***D***	***V***	***S***	KRRSN***K***S	THLK***Y***K	D***S***DQI***S***LYAQA	KCNSEC	VSTKRSQQTVIPNIGSR	NNEKF	KIRSGKS	***S***
A/Mexico/DIF835/2003	D	***S***	R	***D***	***V***	***S***	KRRSN***K***S	THLK***Y***K	D***S***DQI***S***LYAQA	KCNSEC	VSTKRSQQTVIPNIGSR	NNEKF	KIRSGKS	***S***
A/Mexico/DIF940/2003	D	***S***	R	***D***	***V***	***S***	KRRSN***K***S	THLK***Y***K	***N***NDQI***S***LYAQA	KCNSEC	VSTKRSQQTVIPNIGSR	NNEKF	KIRSGKS	***S***
A/California/7/2004	D	***S***	R	***D***	I	P	KRRSNNS	THLK***Y***K	DNDQIFLYAQA	KCNSEC	VSTKRSQQTVIPNIGSR	NNEKF	KIRSGKS	***S***
A/Wisconsin/67/2005	D	F	R	N	I	P	KRRSNNS	T***Q***LKFK	DNDQIFLYAQA	KCNSEC	VSTKRSQQTVIPNIGSR	NNEKF	KIRSGKS	N
A/Brisbane/10/2007	D	F	R	N	I	P	KRRSNNS	THLKFK	DNDQIF***P***YAQA	KCNSEC	VSTKRSQQTVIPNIGSR	NNEKF	KIRSGKS	N
A/Perth/6/2009	D	F	R	N	I	P	KRRSNNS	THL***N***FK	D***K***DQIFLYAQA	KCNSEC	VSTKRSQQTV***S***PNIGSR	NNEK***Q***	KIRSGKS	N
A/Victoria/361/2011	D	F	R	N	I	P	KRRSNNS	T***Q***L***N***FK	D***K***DQIFLYAQA	KCNSEC	VSTKRSQQ***A***VIPNIG***Y***R	NNEK***Q***	KIRSGKS	N
A/Mexico/DIF2112/2005	D	F	R	N	I	P	KRRSNNS	THLKFK	DNDQIFLYAQA	KCNSEC	VSTKRSQQTVIPNIGSR	NNEKF	KIRSGKS	N
A/Mexico/DIF2160/2005	D	F	R	N	I	P	KRRSNNS	THLKFK	DNDQIFLYAQA	KCNSEC	VSTKRSQQTVIPNIGSR	NNEKF	KIRSGKS	N
A/Mexico/DIF2246/2005	D	F	R	N	I	P	KRRSNNS	THLKFK	DNDQIFLYAQA	KCNSEC	VSTKRSQQTVIPNIGSR	NNEKF	KIRSGKS	N
A/Mexico/TLA2227/2005	D	F	R	N	I	P	KRRSNNS	THLKFK	DNDQIFLYAQA	KCNSEC	VSTKRSQQTVIPNIGSR	NNEKF	KIRSGKS	N
A/Mexico/QUE2270/2006	D	F	R	N	I	P	KRRSNNS	THLKFK	DNDQIFLYAQA	KCNSEC	VSTKRSQQTVIPNIGSR	NNEKF	KIRSGKS	N
A/Mexico/DIF2601/2006	D	F	R	N	I	P	KRRSNNS	THLKFK	DNDQIFLYAQA	KCNSEC	VSTKRSQQTVIPNIGSR	NNEKF	KIRSGKS	N
A/Mexico/DIF29/2006	D	F	R	N	I	P	KRRSNNS	THLKFK	DNDQIFLYAQA	KCNSEC	VSTKRSQQTVIPNIGSR	NNEKF	KIRSGKS	N
A/Mexico/MEX2640/2006	D	F	R	N	I	P	KRRSNNS	THLKFK	DNDQIFLYAQA	KCNSEC	VSTKRSQQTVIPN***V***GSR	NNEKF	KIRSGKS	N
A/Mexico/NAY2090/2007	D	F	R	***D***	I	P	KRRSNNS	THLKFK	DNDQIFLYAQA	KCNSEC	VSTKRSQQTVIPN***V***GSR	NNEKF	KIRSGKS	N
A/Mexico/JAL25206/2009	D	F	R	N	I	P	KRRSNNS	T***SH***KFK	DNDQIFLYAQA	KCNSEC	VSTKRSQQTVIPNIGSR	NNEK***E***	KIRSGKS	N
A/Mexico/JAL25216/2009	D	F	R	N	I	P	KRRSNNS	THLKFK	DNDQIFLYAQA	KCNSEC	VSTKRSQQTVIPNIGSR	NNEK***E***	KIRSGKS	N
A/Mexico/VER40/2011	D	F	R	N	I	P	KRRSNNS	THL***N***FK	D***K***DQIFLYAQA	KCNS***A***C	VSTKRSQQ***A***VIPNIGSR	NNEK***Q***	KIRSGKS	N
A/Mexico/VER59/2011	D	F	R	N	I	P	KRRSNNS	THL***N***FK	D***K***DQIFLYAQA	KCNS***A***C	VSTKRSQQ***A***VIPNIGSR	NNEK***Q***	KIRSGKS	N
A/Mexico/VER60/2011	D	F	R	N	I	P	KRRSNNS	THL***N***FK	D***K***DQIFLYAQA	KCNSEC	VSTKRSQQ***A***VIPN***V***GSR	NNEK***Q***	KIRSGKS	N
A/Mexico/VER58/2012	D	F	R	N	I	P	KRRSNNS	THL***N***FK	D***K***DQIFLYAQA	KCNS***A***C	VSTKRSQQ***A***VIPNIGSR	NNEK***Q***	KIRSGKS	N

1 Determined by HA2 numbering (+16 from aminoacid one in coding sequence, [39], [40].

2 Amantadine sensitivity given by mutation in position 31 of M2 protein. S = sensitivity, N = resistance.

3 Antigenic sites B and E are conformational epitopes.

4 Changes of interest are shown in bold and italics.

As expected, more variations were observed in the antigenic sites (Table 2). No changes were found between the antigenic sites of isolate A/Mexico/DIF2662/2003 from clade ‘B’ and the reference strain A/Korea/770/2002 (Table 2). The A/Panama/2007/99 and A/Korea/770/2002 reference strains share the amino acid S in position 189 of antigenic site B, evidence of the similarities between the HA of the Korea viruses and ‘old’ viruses from 2002 or before. Interestingly, the largest number of changes between the between Korea, Fujian and California cluster, was found amongst the Fujian and Korea clusters, with two changes in receptor binding sites 226 and 227 and four changes in antigenic sites (A 145, B 188, B 189 and B 195) (Table 2).

Jin et al. have described that H155T and Q156H are the two amino acid changes responsible for the antigenic drift between Panama-like and Fujian-like viruses, and a total of 13 amino acid differences between these group of viruses can be observed [39]. We searched manually in the HA alignment to find differences among the viruses used in this study and found out that contrasting to the antigenic characterization results, isolate A/Mexico/DIF940/2003 (characterized as A/Panama/2007/99-like) had the Fujian-like markers 155T and 156H and shares only one out of thirteen amino acid markers (V226) with the A/Panama/2007/99 reference strain, with the rest of the markers corresponding to the Fujian viruses. Therefore, we cannot rule out the possibility that antigenic identity of the isolate A/Mexico/DIF940/2003 might not be the correct one. Unfortunately, access to additional information on the antigenicity of the strains (e.g. HI titers) from the CDC was not possible.

Additional incongruences in the distribution of the Panama and Fujian antigenic markers among the A/H3N2 viruses in the dataset used were observed. Marker Q156, specific of A/Panama/2007/99 was also observed in the reference strains A/Wisconsin/67/2005 and A/Victoria/361/2011, while S21 is only observed in the A/Panama/2007/99 strain and no other virus. Markers 128T and 219S are present in all viruses from 1999 to 2013, including A/Panama/2007/99 and A/Mexico/DIF940/2003; with exception of vaccine selected strain A/Wyoming/03/2003. Marker 183L is not present in any virus from the dataset, including A/Panama/2007/99, as all viruses have the Fujian-like 183H (data not shown).

All viruses from the N-lineage were found to have identical amino acid sequences, with only one amino acid change when compared to A/Wisconsin/67/2005 (Table 2). With respect to the viruses belonging to Brisbane cluster, early isolates (A/Mexico/MEX2640/2006 and A/Mexico/NAY2090/2007) have identical antigenic sites, while they showed differences from the 2009 viruses (A/Mexico/JAL25206/2009 and A/Mexico/JAL25216/2009) (Table 2). Changes associated with A/Victoria/361/2011 reference strain were found in the Mexican isolates from 2011, which congruently grouped within the Victoria cluster (Table 1 and 2). Three of these isolates presented an additional change in antigenic site C (196A) (Table 2).

Finally, sequence analysis of the M2 gene revealed that all influenza isolates detected after 2004 in Mexico are amantadine-resistant, having an asparagine in position 31 of the M2 protein, while isolates from 2003 or earlier (belonging to the clade ‘B’ or the Korea cluster) are amantadine-sensitive, having a serine in this position (Table 2).

## Discussion

In this work we analyzed all eight segments of 19 influenza A/H3N2 viruses isolated between 2003 and 2012 in Mexico and compared them to a set of A/H3N2 influenza virus strains isolated in North America from 1998 to 2013. Previous studies report frequent co-circulation of different lineages during single influenza seasons [8], [29]. We observed co-circulation within the 2002–2003 and 2003–2004 seasons (the Korea cluster and clade ‘B’), and within the 2005–2006 season (the N-lineage and Brisbane cluster). The co-circulation of the Korea cluster during the 2003–2004 season is indicated by the presence of A/Mexico/DIF835/2003 and A/NewYork/204/2003 viruses. However, both of these isolates were collected early during the 2003–2004 season (in July and August, respectively), making it difficult to conclude on how “late” from the rest of the 2002–2003 viruses these strains really are.

It has been suggested that influenza A/H3N2 viruses are seeded each year from South-East Asia and disappear at the end of season [10]. However, local persistence of viral lineages and other complex evolutionary patterns for A/H3N2 influenza viruses have also been detected [5], suggesting that strains from North America do contribute to A/H3N2 global evolution [8]. In a global context, numerous reassortment events have been detected among H3N2 circulating in humans, and some of these reassortant variants have been found to persist over time [5], [8], [13], [29], [30], [31]. The differences in clustering of HA and NA gene segments within phylogenetic trees suggest that reassortment can occur relatively frequently over time and it has been demonstrated for a limited number of influenza seasons that multiple lineages of influenza A (H3N2) viruses co-circulate, persist and reassort within the human population [13], [29]. In a local scale, there are also studies that evidence the co-circulation of different lineages of viruses and the occurrence of multiple reassortment events in several countries [42], [43], [44], [45], [46]. In general, we observed that in Mexico, as in North America, during each influenza season virus cluster according to their date of collection rather than by location. There is no evidence for *in situ* evolution of Mexican A/H3N2 viruses, supporting the hypothesis of global circulation of influenza [47].

We found evidence for possible local persistence of some viral lineages in Mexico between different influenza seasons. While persistence of the N-lineage cannot be fully supported, as it is well known that during each flu season there is an overlap in the circulation of lineages from the previous and the starting season, the presence of the Brisbane cluster during three influenza seasons indicates persistence, and not a simple overlap. Nonetheless, our observations on local persistence of lineages within different seasons in Mexico should be further evaluated, as it is difficult to prove persistence within a geographic region without serial sampling of the same location.

We also detected a novel cluster named here as Korea after the reference strain A/Korea/770/2002. However, because there are no other gene segments (apart from HA gene) for the reference strain A/Korea/770/2002 available in the NCBI database, we cannot verify if this strain co-segregate within the Korea cluster in the remaining genes. The A/Fujian/411/2002 and A/Korea/770/2002-like viruses had been characterized as antigenically equivalent [48], [49], but here we have shown that the Fujian cluster is clearly phylogenetically distinct from the smaller Korea cluster. Moreover, we have shown that the Korea cluster had global distribution.

As inferred from our phylogenetic analysis, the Korea cluster shows a complex genetic pattern suggesting that it may have originated by extensive reassortment. The HA and PB1 of the Korea viruses are related to ‘old viruses’ from 1999–2000 (Panama cluster) and from 2002–2003 (Fujian cluster and to clade ‘B’), while the NA, PB2, PA and NP genes are basal to the main tree trunk from which strains collected after 2004 diverge, related either to the California cluster or to the N-lineage. With respect to the other lineages known to have circulated in North America, we did not observe any Mexican isolates belonging to the Fujian, California, or Perth cluster, but such observations could be a result of the low number of samples used or sampling bias.

Changes were observed in antigenic sites in the HA protein among our isolates and reference strains, but no clear variation pattern associated with antigenic/phylogenetic groups could be detected in viruses collected within a short temporal range (with 6 months to one year). Clear differences could be made between viruses collected within a larger spatial range (from before 2003 and after 2004), given mainly by changes in receptor binding sites. Since the emergence of the N-lineage in 2005, no frequent changes in the receptor binding sites have been detected, suggesting that receptor binding sites are under functional constrain, and therefore only few changes are fixed in the main viral population.

In conclusion, our findings show that different A/H3N2 viral lineages in Mexico can co-circulate and can also persist locally in between different influenza seasons and thus contributing to the local evolution of A/H3N2 viruses. A novel minor cluster was also identified, named here as Korea, that circulated worldwide during 2003. Our work contributes with useful information on the epidemiological and evolutionary behavior of A/H3N2 viruses in Mexico that can be integrated with global data.

## Supporting Information

Figure S1 **Prevalent A/H3N2 antigenic groups circulating in human populations from 2000 to 2012.** Schematic representation of predominant antigenic groups that circulated since 2000 as reported [16], [17], [18], [19], [20], [21], [22], [23], [24], [25], [26], [27], [28]. Time line is shown at the top. S31N points to introduction of amantadine resistance mutation in circulating strains, with broken line representing amantadine sensitive strains and continuous line represents presence of resistant viruses [31]. Reference strains of antigenic groups detected are shown.
(TIF)

Figure S2 **Phylogenetic analysis based on the coding sequence of the PB2 gene of 19 samples isolated in Mexico from 2003 to 2012.** ML tree for the PB2 viral gene with a background of 756 selected human A/H3N2 influenza viruses from North America. Support values were determined by aLRT and only values ≥0.70 are shown for significant nodes. The tree is mid-point rooted for purposes of clarity, and all horizontal branches are drawn to scale. The position of the Mexican isolates in the tree is indicated by coding letters in blue, while the position of reference strains is indicated by numbers in red, as depicted in the left portion of figure. Branches are colored according strain names: blue branches indicate Mexican isolates, while red branches indicate reference strains. The rest of the viruses are shown black.
(TIF)

Figure S3 **Phylogenetic analysis based on the coding sequence of the PB1 gene of 19 samples isolated in Mexico from 2003 to 2012.** ML tree for the PB1 viral gene with a background of 759 selected human A/H3N2 influenza viruses from North America. Support values were determined by aLRT and only values ≥0.70 are shown for significant nodes. The tree is mid-point rooted for purposes of clarity, and all horizontal branches are drawn to scale. The position of the Mexican isolates in the tree is indicated by coding letters in blue, while the position of reference strains is indicated by numbers in red, as depicted in the left portion of figure. Branches are colored according strain names: blue branches indicate Mexican isolates, while red branches indicate reference strains. The rest of the viruses are shown black.
(TIF)

Figure S4 **Phylogenetic analysis based on the coding sequence of the PA gene of 19 samples isolated in Mexico from 2003 to 2012.** ML tree for the PA viral gene with a background of 752 selected human A/H3N2 influenza viruses from North America. Support values were determined by aLRT and only values ≥0.70 are shown for significant nodes. The tree is mid-point rooted for purposes of clarity, and all horizontal branches are drawn to scale. The position of the Mexican isolates in the tree is indicated by coding letters in blue, while the position of reference strains is indicated by numbers in red, as depicted in the left portion of figure. Branches are colored according strain names: blue branches indicate Mexican isolates, while red branches indicate reference strains. The rest of the viruses are shown black.
(TIF)

Figure S5 **Phylogenetic analysis based on the coding sequence of the NP gene of 19 samples isolated in Mexico from 2003 to 2012.** ML tree for the NP viral gene with a background of 627 selected human A/H3N2 influenza viruses from North America. Support values were determined by aLRT and only values ≥0.70 are shown for significant nodes. The tree is mid-point rooted for purposes of clarity, and all horizontal branches are drawn to scale. The position of the Mexican isolates in the tree is indicated by coding letters in blue, while the position of reference strains is indicated by numbers in red, as depicted in the left portion of figure. Branches are colored according strain names: blue branches indicate Mexican isolates, while red branches indicate reference strains. The rest of the viruses are shown black.
(TIF)

Figure S6 **Phylogenetic analysis based on the coding sequence of the M gene of 19 samples isolated in Mexico from 2003 to 2012.** ML tree for the M viral gene with a background of 461 selected human A/H3N2 influenza viruses from North America. Support values were determined by aLRT and only values ≥0.70 are shown for significant nodes. The tree is mid-point rooted for purposes of clarity, and all horizontal branches are drawn to scale. The position of the Mexican isolates in the tree is indicated by coding letters in blue, while the position of reference strains is indicated by numbers in red, as depicted in the left portion of figure. Branches are colored according strain names: blue branches indicate Mexican isolates, while red branches indicate reference strains. The rest of the viruses are shown black.
(TIF)

Figure S7 **Phylogenetic analysis based on the coding sequence of the NS gene of 19 samples isolated in Mexico from 2003 to 2012.** ML tree for the NS viral gene with a background of 414 selected human A/H3N2 influenza viruses from North America. Support values were determined by aLRT and only values ≥0.70 are shown for significant nodes. The tree is mid-point rooted for purposes of clarity, and all horizontal branches are drawn to scale. The position of the Mexican isolates in the tree is indicated by coding letters in blue, while the position of reference strains is indicated by numbers in red, as depicted in the left portion of figure. Branches are colored according strain names: blue branches indicate Mexican isolates, while red branches indicate reference strains. The rest of the viruses are shown black.
(TIF)

Figure S8 **Phylogenetic analysis based on the coding sequence of the HA gene of 804 viruses circulating worldwide from 2002 to 2004.** ML tree for the HA viral gene of globally circulating human A/H3N2 influenza viruses collected from 2002 to 2004. Support values were determined by aLRT and only values ≥0.70 are shown for significant nodes. The tree is mid-point rooted for purposes of clarity, and all horizontal branches are drawn to scale. The clusters of importance were highlighted with different colors, using magenta for the Korea cluster, green for the Clade ‘B’, dark blue for the Fujian cluster and light blue for the California cluster. Highlighted in black are viruses found within the Korea cluster previously in the North American HA tree.
(TIF)

Figure S9 **Phylogenetic analysis based on the coding sequence of the NA gene of 660 viruses circulating worldwide from 2002 to 2004.** ML tree for the NA viral gene of globally circulating human A/H3N2 influenza viruses collected from 2002 to 2004. Support values were determined by aLRT and only values ≥0.70 are shown for significant nodes. The tree is mid-point rooted for purposes of clarity, and all horizontal branches are drawn to scale. The clusters of importance were highlighted with different colors, using magenta for the Korea cluster, green for the Clade ‘B’, dark blue for the Fujian cluster and light blue for the California cluster. Highlighted in black are viruses found within the Korea cluster previously in the North American NA tree.
(TIF)
