# Supplementary figures and images for: Molecular Epidemiology of Influenza A/H3N2 Viruses Circulating in Mexico from 2003 to 2012
Source: PLoS One. 2014 Jul 30;9(7):e102453. doi: 10.1371/journal.pone.0102453 (PMC4116128; doi:10.1371/journal.pone.0102453)

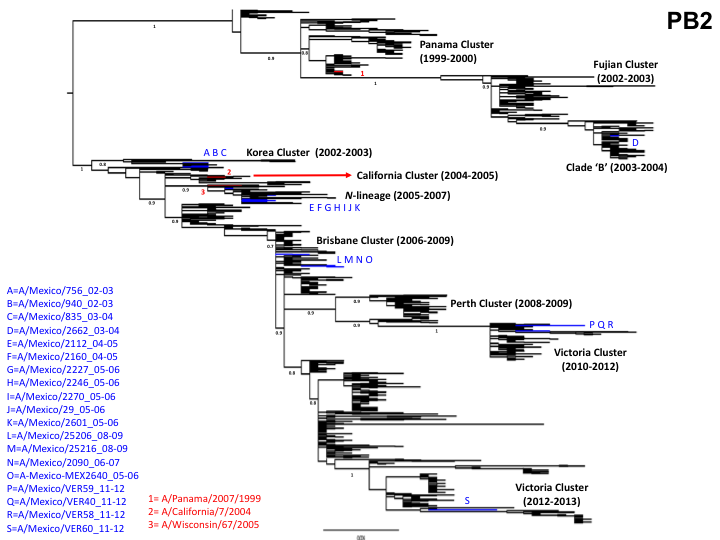

Supplement: Figure S2 — Phylogenetic analysis based on the coding sequence of the PB2 gene of 19 samples isolated in Mexico from 2003 to 2012. ML tree for the PB2 viral gene with a background of 756 selected human A/H3N2 influenza viruses from North America. Support values were determined by aLRT and only values ≥0.70 are shown for significant nodes. The tree is mid-point rooted for purposes of clarity, and all horizontal branches are drawn to scale. The position of the Mexican isolates in the tree is indicated by coding letters in blue, while the position of reference strains is indicated by numbers in red, as depicted in the left portion of figure. Branches are colored according strain names: blue branches indicate Mexican isolates, while red branches indicate reference strains. The rest of the viruses are shown black. (TIF) [file pone.0102453.s002.tif]

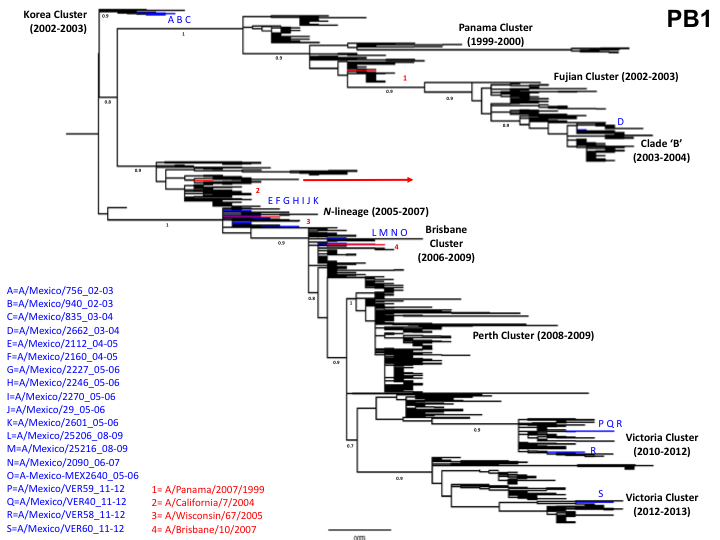

Supplement: Figure S3 — Phylogenetic analysis based on the coding sequence of the PB1 gene of 19 samples isolated in Mexico from 2003 to 2012. ML tree for the PB1 viral gene with a background of 759 selected human A/H3N2 influenza viruses from North America. Support values were determined by aLRT and only values ≥0.70 are shown for significant nodes. The tree is mid-point rooted for purposes of clarity, and all horizontal branches are drawn to scale. The position of the Mexican isolates in the tree is indicated by coding letters in blue, while the position of reference strains is indicated by numbers in red, as depicted in the left portion of figure. Branches are colored according strain names: blue branches indicate Mexican isolates, while red branches indicate reference strains. The rest of the viruses are shown black. (TIF) [file pone.0102453.s003.tif]

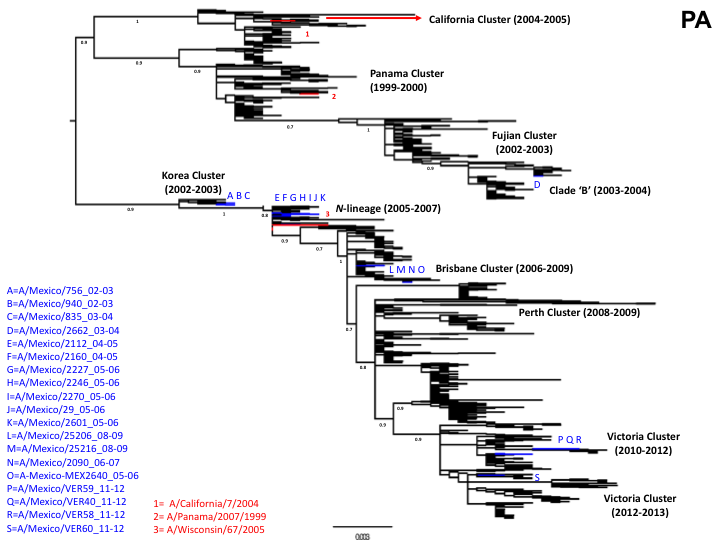

Supplement: Figure S4 — Phylogenetic analysis based on the coding sequence of the PA gene of 19 samples isolated in Mexico from 2003 to 2012. ML tree for the PA viral gene with a background of 752 selected human A/H3N2 influenza viruses from North America. Support values were determined by aLRT and only values ≥0.70 are shown for significant nodes. The tree is mid-point rooted for purposes of clarity, and all horizontal branches are drawn to scale. The position of the Mexican isolates in the tree is indicated by coding letters in blue, while the position of reference strains is indicated by numbers in red, as depicted in the left portion of figure. Branches are colored according strain names: blue branches indicate Mexican isolates, while red branches indicate reference strains. The rest of the viruses are shown black. (TIF) [file pone.0102453.s004.tif]

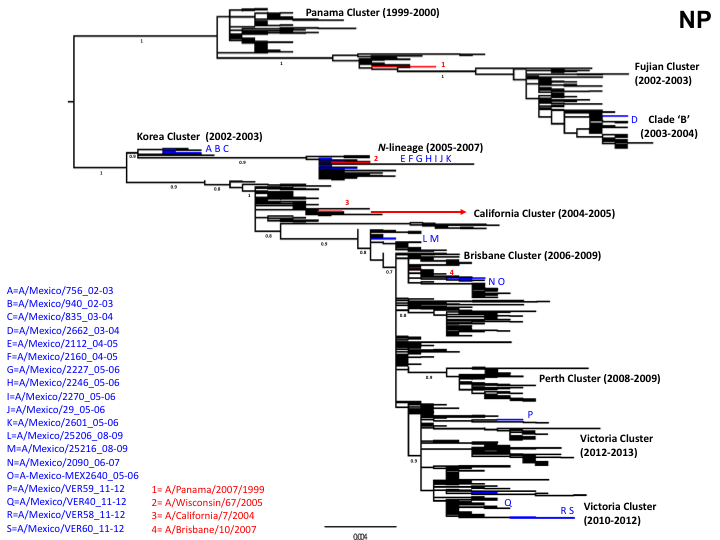

Supplement: Figure S5 — Phylogenetic analysis based on the coding sequence of the NP gene of 19 samples isolated in Mexico from 2003 to 2012. ML tree for the NP viral gene with a background of 627 selected human A/H3N2 influenza viruses from North America. Support values were determined by aLRT and only values ≥0.70 are shown for significant nodes. The tree is mid-point rooted for purposes of clarity, and all horizontal branches are drawn to scale. The position of the Mexican isolates in the tree is indicated by coding letters in blue, while the position of reference strains is indicated by numbers in red, as depicted in the left portion of figure. Branches are colored according strain names: blue branches indicate Mexican isolates, while red branches indicate reference strains. The rest of the viruses are shown black. (TIF) [file pone.0102453.s005.tif]

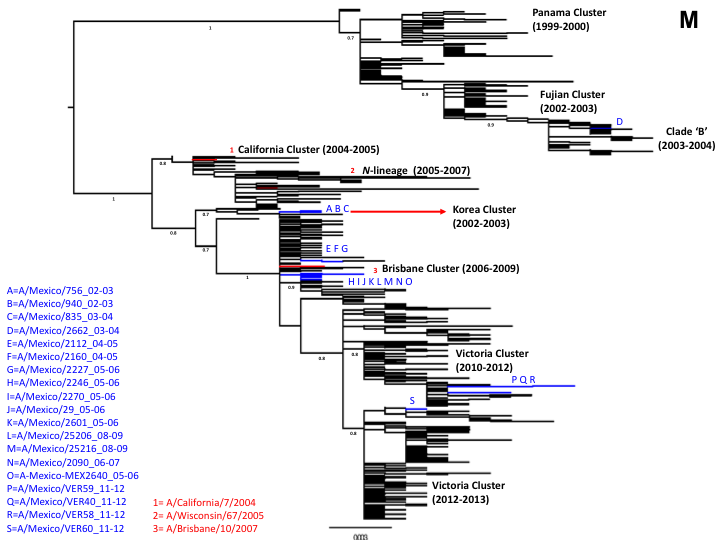

Supplement: Figure S6 — Phylogenetic analysis based on the coding sequence of the M gene of 19 samples isolated in Mexico from 2003 to 2012. ML tree for the M viral gene with a background of 461 selected human A/H3N2 influenza viruses from North America. Support values were determined by aLRT and only values ≥0.70 are shown for significant nodes. The tree is mid-point rooted for purposes of clarity, and all horizontal branches are drawn to scale. The position of the Mexican isolates in the tree is indicated by coding letters in blue, while the position of reference strains is indicated by numbers in red, as depicted in the left portion of figure. Branches are colored according strain names: blue branches indicate Mexican isolates, while red branches indicate reference strains. The rest of the viruses are shown black. (TIF) [file pone.0102453.s006.tif]

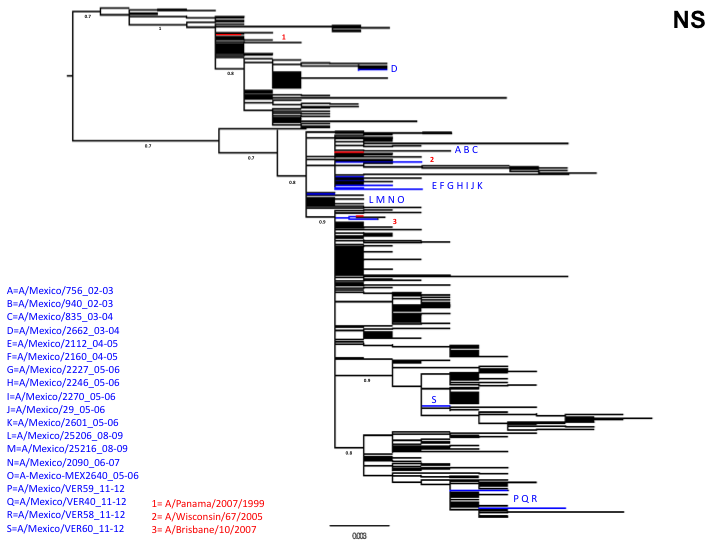

Supplement: Figure S7 — Phylogenetic analysis based on the coding sequence of the NS gene of 19 samples isolated in Mexico from 2003 to 2012. ML tree for the NS viral gene with a background of 414 selected human A/H3N2 influenza viruses from North America. Support values were determined by aLRT and only values ≥0.70 are shown for significant nodes. The tree is mid-point rooted for purposes of clarity, and all horizontal branches are drawn to scale. The position of the Mexican isolates in the tree is indicated by coding letters in blue, while the position of reference strains is indicated by numbers in red, as depicted in the left portion of figure. Branches are colored according strain names: blue branches indicate Mexican isolates, while red branches indicate reference strains. The rest of the viruses are shown black. (TIF) [file pone.0102453.s007.tif]

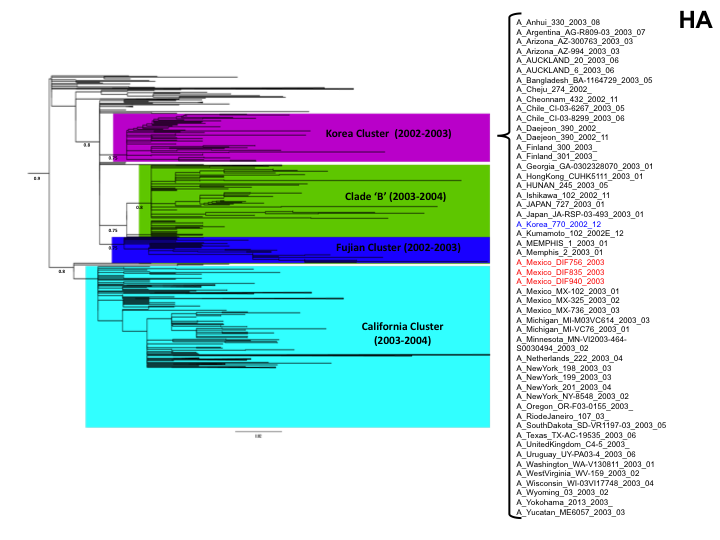

Supplement: Figure S8 — Phylogenetic analysis based on the coding sequence of the HA gene of 804 viruses circulating worldwide from 2002 to 2004. ML tree for the HA viral gene of globally circulating human A/H3N2 influenza viruses collected from 2002 to 2004. Support values were determined by aLRT and only values ≥0.70 are shown for significant nodes. The tree is mid-point rooted for purposes of clarity, and all horizontal branches are drawn to scale. The clusters of importance were highlighted with different colors, using magenta for the Korea cluster, green for the Clade ‘B’, dark blue for the Fujian cluster and light blue for the California cluster. Highlighted in black are viruses found within the Korea cluster previously in the North American HA tree. (TIF) [file pone.0102453.s008.tif]

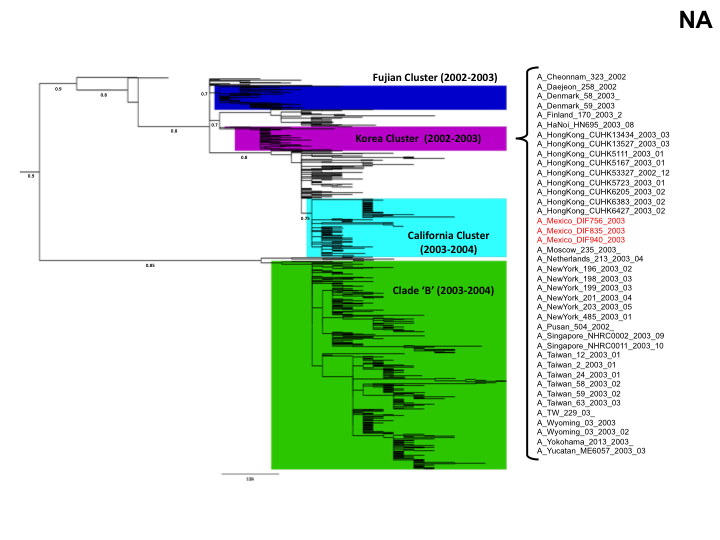

Supplement: Figure S9 — Phylogenetic analysis based on the coding sequence of the NA gene of 660 viruses circulating worldwide from 2002 to 2004. ML tree for the NA viral gene of globally circulating human A/H3N2 influenza viruses collected from 2002 to 2004. Support values were determined by aLRT and only values ≥0.70 are shown for significant nodes. The tree is mid-point rooted for purposes of clarity, and all horizontal branches are drawn to scale. The clusters of importance were highlighted with different colors, using magenta for the Korea cluster, green for the Clade ‘B’, dark blue for the Fujian cluster and light blue for the California cluster. Highlighted in black are viruses found within the Korea cluster previously in the North American NA tree. (TIF) [file pone.0102453.s009.tif]
